# Defining the Species *Micromonospora saelicesensis* and *Micromonospora noduli* Under the Framework of Genomics

**DOI:** 10.3389/fmicb.2018.01360

**Published:** 2018-06-25

**Authors:** Raúl Riesco, Lorena Carro, Brenda Román-Ponce, Carlos Prieto, Jochen Blom, Hans-Peter Klenk, Philippe Normand, Martha E. Trujillo

**Affiliations:** ^1^Departament of Microbiology and Genetics, Edificio Departamental, University of Salamanca, Salamanca, Spain; ^2^Servicio de Bioinformática, NUCLEUS, Edificio I+D+i, University of Salamanca, Salamanca, Spain; ^3^Bioinformatics and Systems Biology, Justus-Liebig-University Giessen, Giessen, Germany; ^4^School of Natural and Environmental Sciences, Newcastle University, Newcastle upon Tyne, United Kingdom; ^5^Centre National de la Recherche Scientifique-UMR5557 Ecologie Microbienne, Université de Lyon, Université Lyon1, Villeurbanne, France

**Keywords:** *Micromonospora*, genome sequencing, phylogenomic analysis, nitrogen-fixing nodule, taxonomy, species delimitation

## Abstract

The type isolates of species *Micromonospora saelicesensis* and *Micromonospora noduli* are Gram-stain positive actinobacteria that were originally isolated from nitrogen fixing nodules of the legumes *Lupinus angustifolius* and *Pisum sativum*, respectively. These two species are very closely related and questions arise as to whether they should be merged into a single species. To better delineate the relationship of *M. saelicesensis* and *M. noduli*, 10 strains isolated from plant tissue (nodules and leaves) and identified by their 16S rRNA gene sequences as either *M. saelicensesis* or *M. noduli*, based on a cut-off value of ≥99.5% were selected for whole-genome sequencing and compared with the type strains of *M. saelicesensis* Lupac 09^T^ and *M. noduli* GUI43^T^ using overall genome relatedness indices (OGRI) which included ANI, OrthoANI and digital DNA-DNA hybridization. Whole- and core-genome phylogenomic analyses were also carried out. These results were compared with the topologies of the 16S rRNA and *gyrB* gene phylogenies. Good correlation was found between all trees except for the 16S rRNA gene. Overall results also supported the current classification of *M. saelicesensis* and *M. noduli* as separate species. Especially useful was the core-genome phylogenetic analyses based on 92 genes and the dDDH results which were highly correlated. The importance of using more than one strain for a better definition of a species was also shown. A series of *in vitro* phenotypic assays performed at different times were compared with *in silico* predictions based on genomic data. *In vitro* phenotypic tests showed discrepancies among the independent studies, confirming the lack of reproducibility even when tests were performed in the same laboratory. On the other hand, the use of *in silico* predictions proved useful for defining a stable phenotype profile among the strains analyzed. These results provide a working framework for defining *Micromonospora* species at the genomic and phenotypic level.

## Introduction

To define a species, current prokaryotic taxonomy integrates multiple aspects of a microorganism that include phenotypic and genotypic data (Chun and Rainey, 2014). This approach, known as polyphasic taxonomy (Colwell, 1970; Vandamme et al., 1996) has contributed for several decades to improve classification and identification schemes, however, its limitations and pitfalls, particularly in relation to reproducibility of some methods and/or the difficulty of data storage have been timely addressed (Sutcliffe et al., 2012; Vandamme and Peeters, 2014; Thompson et al., 2015).

The introduction and improvement of cost-effective whole-genome sequencing methods provide a new working framework. Unlike DNA-DNA hybridization, that was heralded as the “golden standard” for defining genomic species in 1987 (Wayne et al., 1987), genomic data can be stored and made available to the scientific community for subsequent comparisons (Chun and Rainey, 2014). Furthermore, genomic data can also be used to predict phenotypic traits which can then be tested in the laboratory, reducing the need to perform labor-intensive and non-repoducible tests (Sutcliffe et al., 2012; Amaral et al., 2014).

Another problem is the definition of species based on single-strain representatives. This approach does not allow the recognition of intra-species diversity and limits the proposal for a sound and testable definition of a prokaryotic species. While the number of genomes representing bacterial species, in most cases, include only the type strain, it is also necessary to study several members within the same species to better understand intraspecies variation. Unfortunately, for most species, only one strain (the type strain) has been described and single-strain descriptions hinder the possibility for such studies.

The genus *Micromonospora* represented by Gram-stain positive, filamentous and sporulating actinobacteria, belongs to the family *Micromonosporaceae* of the order *Micromonosporales* in the phylum *Actinobacteria* (Genilloud, 2015a,b,c). The type species of the genus is *Micromonospora chalcea* and currently includes 81 species with validly published names http://www.bacterio.net/micromonospora.html (Parte, 2014). Most of these species have been described in the past 10 years with representative strains isolated from diverse habitats such as soil (Li and Hong, 2016; Lee and Whang, 2017), aquatic habitats (Trujillo et al., 2005; de Menezes et al., 2012), plant tissues (Carro et al., 2012, 2013; Kittiwongwattana et al., 2015; Trujillo et al., 2015; Kaewkla et al., 2017) and other environments (Hirsch et al., 2004; Nimaichand et al., 2013; Lin et al., 2015). Recently, a revised classification of the genus *Micromonospora* based on genome sequence data has been proposed (Carro et al., 2018).

In 2007, three strains recovered from internal nodular tissue of the plant *Lupinus angustifolius* were formally described as *Micromonospora saelicesensis* (Trujillo et al., 2007). Recent studies have shown that this species is widely distributed in legumes (e.g., *Trifolium, Lupinus, Pisum*, etc.), especially in nodules (Trujillo et al., 2015). *Micromonospora noduli* described as a single representative, strain GUI43^T^, isolated from the nodular tissue of *Pisum sativum* was found to be closely related to *M. saelicesensis* (Carro et al., 2016). While the DNA-DNA hybridization value of 63.4% (62.3 reciprocal) is below the accepted threshold of 70% (Wayne et al., 1987) these two species share many features, and the question arises whether they should be merged into a single species. Therefore, this study was designed to determine the level of taxonomic relationship between ten strains initially identified as *M. saelicesensis* or *M. noduli* using a 16S rRNA gene sequence comparison with a similarity threshold of ≥99.5%. Draft whole-genome sequences were obtained for all strains, including the type strain *M. noduli* GUI43^T^ and data was analyzed using a combination of overall genome related indices (OGRI) and phylogenomic analyses. Furthermore, to obtain information about intra-species variation, especially at the phenotypic level, these studies were complemented with physiological and biochemical data. The integration of all these studies support the current status of *M. saelicesensis* and *M. noduli* as different species and the approach presented in this work provides a good method for the definition of species in the genus *Micromonospora*.

## Materials and methods

### Isolation of strains

The list of *Micromonospora* strains used in this study is given in Table 1. All strains, except for LAH08, were isolated from nitrogen fixing nodules of five different legumes between 2003 and 2015 as described previously (Trujillo et al., 2010). Isolation of strain LAH08 from the leaves of *L. angustifolius* was done after surface sterilization of the plant material by immersing in 70% ethanol (v/v) for 1 min, transferred to 3.5% w/v sodium hypochlorite solution for 2 min and rinsed five times with sterile distilled water. Sample was crushed with a sterile homogenizing pestle in a microtube and the resulting slurry plated onto yeast extract-humic acid agar (de la Vega, 2010).

**Table 1:** Source of strains used in this study.

**Strain**	**Host plant**	**Isolation**	**Plant collection site**	**Geographical coordinates**	**16S rRNA gene sequence identity**		**References**
Lupac 09^T^	*Lupinus angustifolius*	Nodule	Saelices	40° 40' 06” N; 6° 38' 02” W	*M. saelicesensis* Lupac09^T^	100%	Trujillo et al., 2007
					*M. noduli* GUI43^T^	99.2%	
GAR05	*Cicer arietinum*	Nodule	Cabrerizos	40° 58' 43” N; 5° 36' 46” W	*M. saelicesensis* Lupac09^T^	99.9%	This study
					*M. noduli* GUI43^T^	99.8%	
GAR06	*C. arietinum*	Nodule	Cabrerizos	40° 58' 43” N; 5° 36' 46” W	*M. saelicesensis* Lupac09^T^	99.9%	This study
					*M. noduli* GUI43^T^	99.9%	
Lupac 06	*L. angustifolius*	Nodule	Saelices	40° 40' 06” N; 6° 38' 02” W	*M. saelicesensis* Lupac09^T^	99.9%	Trujillo et al., 2007
					*M. noduli* GUI43^T^	99.7%	
PSN01	*Pisum sativum*	Nodule	Salamanca	40°58′07″ N; 5°39′49″ W	*M. saelicesensis* Lupac09^T^	99.9%	This study
					*M. noduli* GUI43^T^	99.6%	
PSN13	*P. sativum*	Nodule	Salamanca	40°58′07″ N; 5°39′49″ W	*M. saelicesensis* Lupac09^T^	99.9%	This study
					*M. noduli* GUI43^T^	99.8%	
GUI43^T^	*P. sativum*	Nodule	Cañizal	41° 10' 04” N; 5° 22' 08” W	*M. noduli* GUI43^T^	100%	Carro et al., 2016
					M*. saelicesensis* Lupac09^T^	99.2%	
LAH08	*L. angustifolius*	Leaf	Cabrerizos	40° 58' 43” N; 5° 36' 46” W	*M. noduli* GUI43^T^	99.9%	This study
					*M. saelicesensis* Lupac09^T^	99.9%	
Lupac 07	*L. angustifolius*	Nodule	Saelices	40° 40' 06” N; 6° 38' 02” W	*M. noduli* GUI43^T^	99.8%	Trujillo et al., 2007
					*M. saelicesensis* Lupac09^T^	99.7%	
MED15	*Medicago* sp.	Nodule	Salamanca	40°58′07″ N; 5°39′49″ W	*M. noduli* GUI43^T^	100%	This study
					*M. saelicesensis* Lupac09^T^	99.2%	
ONO23	*Ononis* sp.	Nodule	Cabrerizos	40° 58' 43” N; 5° 36' 46” W	*M. noduli* GUI43^T^	100%	This study
					*M. saelicesensis* Lupac09^T^	99.8%	
ONO86	*Ononis* sp.	Nodule	Cabrerizos	40° 58' 43” N; 5° 36' 46” W	*M. noduli* GUI43^T^	99.9%	This study
					*M. saelicesensis* Lupac09^T^	99.8%	

### 16S rRNA gene sequencing and analysis

DNA extraction (REDExtract-N-Amp Plant PCR kit [Sigma]), 16S rRNA gene amplification and sequencing were carried out as previously explained (Trujillo et al., 2010). Assembled sequences were compared against the Ezbiocloud Database (Yoon et al., 2017) and other public platforms (Genbank, EMBL, etc.), and aligned with ClustalX2 (Thompson et al., 1997). *Catellatospora citrea* IMSNU 22008^T^, a member of the family *Micromonosporaceae* was used as outgroup.

Phylogenetic analyses were performed using MEGA (v 7.0.14) (Kumar et al., 2016); distances were calculated with the Kimura 2-parameter and tree topologies were based on the Maximum Likelihood algorithm (Felsenstein, 1981). Total analysis included 1318 positions and a bootstrap (Felsenstein, 1985) sampling of 1,000.

### Whole-genome sequencing, assembly, and annotation

DNA was isolated from 1 g of bacterial cultures grown in ISP 2 broth (Shirling and Gottlieb, 1966) at 28°C for 5–7 days. Cell lysis was done in 5 ml EC buffer containing 60 μl of lysozyme (300 mg/ml, Sigma-Aldrich, USA) and 50 μl of mutanolysin (1,000 U/ml), and incubated at 37°C for 90 min. Five ml of 2% SDS (w/v) and 200 μl of proteinase k (10 mg/ml) were added with gentle mixing for protein precipitation and incubated at 55°C for 3 h. Samples were extracted with phenol:chloroform:isoamyl alcohol (25:24:1 v/v), treated with 35 μl of RNAse A (10 mg/ml) and precipitated with 70% ethanol. Draft genome sequences were determined by MiSeq (300 bp paired end) (Chunlab, Inc.). Libraries were prepared using TruSeq DNA LT Sample Prep kit (Illumina, San Diego, CA, USA) for the Illumina system (Chunlab, Inc.). Illumina sequencing data were assembled with SPAdes 3.10.1 (Algorithmic Biology Lab, St. Petersburg Academic University of the Russian Academy of Sciences). Protein-coding sequences (CDSs) were predicted by Prodigal 2.6.2 (Hyatt et al., 2010). Genes coding for tRNA were searched using tRNAscan-SE 1.3.1 (Schattner et al., 2005). The rRNA and other non-coding RNAs were searched by a covariance model search with Rfam 12.0 database (Nawrocki et al., 2016). All genomes were functionally annotated using the new eggNOG-mapper (Huerta-Cepas et al., 2017) with HMMER mapping mode against actNOG and bacterial HMM databases using all Orthologs. To confirm annotation, the predicted CDSs were compared with Swissprot (Bateman et al., 2015), KEGG (Kanehisa et al., 2014), and SEED (Overbeek et al., 2005) databases using UBLAST program (Edgar, 2010). Principal component analysis was carried out with the COG data using ggfortify v 0.4.3 R package (Tang et al., 2016). Clustering was inferred with K-means clustering algorithm using cluster R package v 2.0.7-1 (Maechler et al., 2018).

CRISPR elements were retrieved using the online application CRISPR-finder, available in http://crispr.i2bc.paris-saclay.fr (Grissa et al., 2007) using default parameters. EDGAR 2.0 platform (Blom et al., 2016) was used to calculate the core genome, dispensable genome and singleton genes.

### gyrB gene phylogeny

*gyrB* nucleotide gene sequences extracted from whole genome sequence data or downloaded from the public databases were used to construct a Maximum-Likelihood phylogenetic tree based on Kimura 2-parameter, using 1001 nucleotide positions and a bootstrap value of 1000. *Catellatospora citrea* DSM 44097^T^, a member of the family *Micromonosporaceae* was used as outgroup.

### OGRI analyses

Average Nucleotide Identity (ANI) (Goris et al., 2007) and OrthoANI (Lee et al., 2016) comparisons were made with the Orthologous Average Nucleotide Identity Tool (OAT) v0.93 https://www.ezbiocloud.net/tools/orthoani. Digital DNA-DNA hybridizations (dDDH) and G+C content differences were obtained with Genome to Genome Distance Calculator (GGDC) v2.0 available at https://ggdc.dsmz.de/ggdc.php# using the recommended settings (Meier-Kolthoff et al., 2013b). A dDDH heatmap was constructed using ComplexHeatmap R package v 1.17.1 (Gu et al., 2016).

### Whole-genome phylogenomic analyses

Genome Blast Distance Phylogeny (GBDP) was used to calculate the intergenomic distances based on whole proteomes (Meier-Kolthoff et al., 2013b). Calculation of a distance matrix was done using the on-line GGDC server, with BLAST+ and recommended formula 2 (optimized for draft genome sequences) (Meier-Kolthoff et al., 2013b). Phylogenetic trees were constructed with FastMe tool (Lefort et al., 2015). Genome sequence accession numbers are provided in Table S1.

### UBCG phylogenomic analysis

Ninety-two bacterial core genes based on the Up to date Bacterial Core Gene (UBCG) tool, https://www.ezbiocloud.net/tools/ubcg were used for phylogenomic tree reconstruction using default parameters (Na et al., 2018). The selection of the representative genes was based on 1429 complete genome sequences, covering 28 phyla and providing a set of genes present in the majority of the genomes or highly conserved single copy genes (Na et al., 2018).

### Physiology

A set of physiological and biochemical tests reported to differentiate between *M. saelicesensis* and *M. noduli* were carried out; these included carbon source utilization, determination of enzymatic activity, NaCl and pH tolerance, temperature range growth and degradation of starch, Tween 20, Tween 80, tyrosine, and urea (Carro et al., 2016). All tests were done in triplicate.

Several carbon sources (19) were tested *in vitro* at different times in the laboratory (2016 and 2017) and compared with the results of the original description of *M. saelicesensis* to check for reproducibility (Trujillo et al., 2007). Draft-genome data was screened for genes coding for proteins for carbon metabolism of the carbon sources assayed.

### Biolog characterization

To generate phenotypic fingerprints of 71 carbon source utilization and 23 chemical sensitivity assays, the strains were tested at 28°C using GEN III Microplates in an Omnilog device (BIOLOG Inc., Hayward, CA, USA). The reference strains *Micromonospora saelicesensis* Lupac 09^T^ and *Micromonospora noduli* GUI43^T^ were included for parallel comparison. One week old cells were suspended in an inoculating fluid (IF C) provided by the manufacturer and inoculated in the GEN III Microplates at a cell density of 80% transmittance. Phenotype microarray mode was used to measure respiration rates yielding a total running time of 7 days using two independent replicates for each strain. Data were recovered and analyzed using the opm package for R, v.1.0.6 (Vaas et al., 2012, 2013). Clustering analyses of the phenotypic microarrays were constructed using the pvclust package for R v.1.2.2 (Suzuki and Shimodaira, 2015). Distinct behaviors between the two repetitions in the reactions were regarded as ambiguous.

## Results

### 16S rRNA gene sequence analysis

The 16S rRNA gene sequences were used to determine the nearest phylogenetic neighbors based on overall sequence similarity in relation to currently described *Micromonospora* species. In all cases, the closest species were *M. saelicesensis* and *M. noduli* with similarity values of 99.2–100% (Table 1).

**Figure 1:**
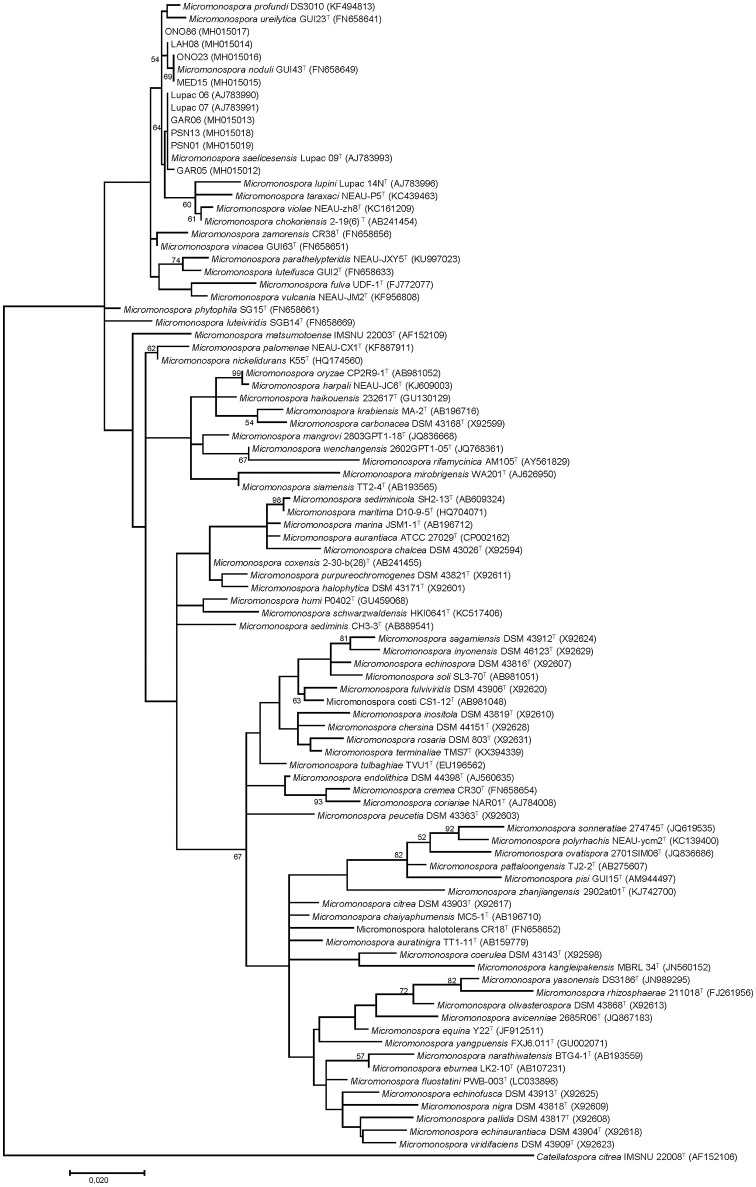
Maximum-likelihood phylogenetic tree based on 16S rRNA gene sequences showing the relationships between 81 *Micromonospora* type and the study strains. Distances were calculated with the Kimura 2-parameter. The tree is based on 1,318 nt. Bootstrap percentages ≥50% (1,000 samplings) are shown at nodes. Bar, 0.02 substitutions per nucleotide.

A phylogenetic tree constructed with the new sequences and those of 81 *Micromonospora* species described to date, distributed the 10 strains into two clusters: Group I contained the type strain *M. saelicesensis* Lupac 09^T^ and the isolates PSN13, GAR06, PSN01, Lupac 06, GAR05, and Lupac 07. Group II was formed with ONO 86, ONO23, LAH08, and MED15 and *M. noduli* GUI43^T^ (Figure 1). The topology clearly showed the close relationship between the two groups as visualized by the branch lengths which were almost inexistent. Group II (*M. noduli*) also showed a close relationship with the type strains of *Micromonospora profundi* isolated from a deep marine sediment (Veyisoglu et al., 2016) and *Micromonospora ureilytica* isolated from *Pisum sativum* (Carro et al., 2016), also recovered in this group. Reported DDH values between *M. saelicesensis* and *M. ureilytica* and *M. profundi* (Veyisoglu et al., 2016) were 28.4 and 56.9%, respectively. A DDH value of 50.9% was found between *M. noduli* GUI 43^T^ and *M. ureilytica* GUI23^T^ (Carro et al., 2016).

### *gyrB* gene phylogeny

**Figure 2:**
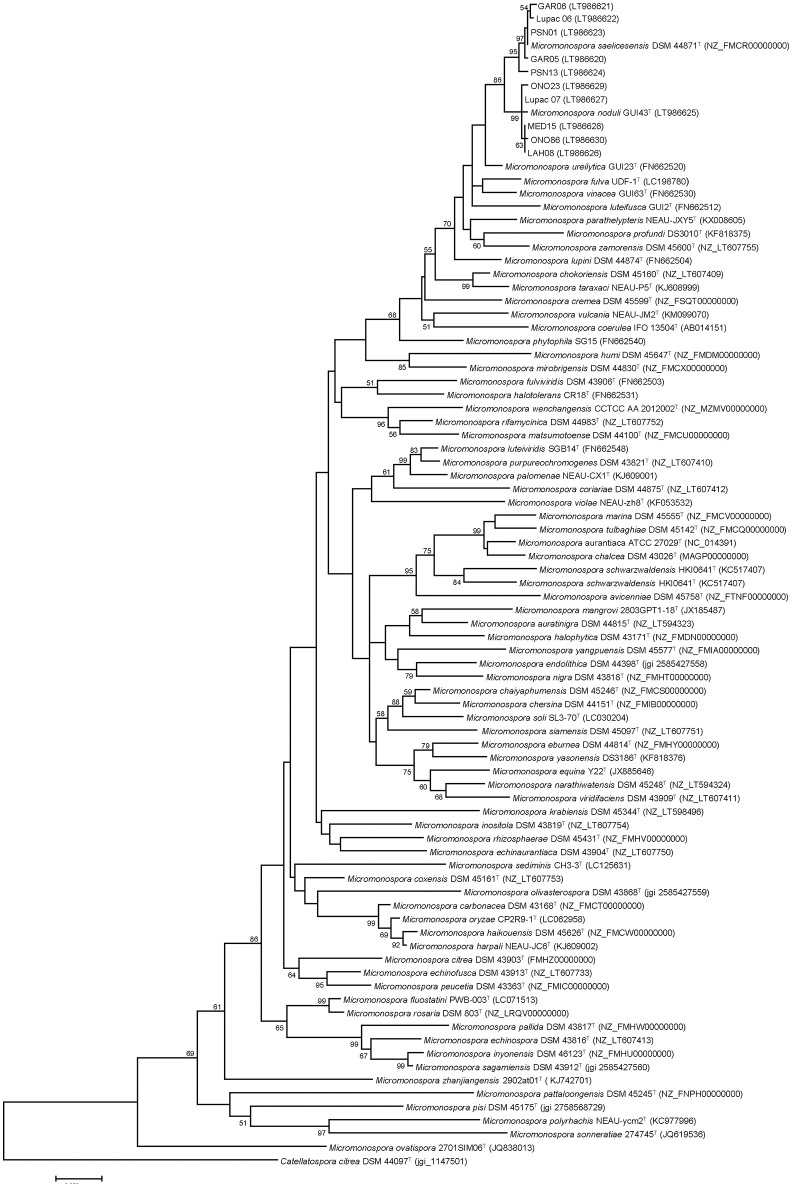
Maximum-likelihood phylogenetic tree based on *gyr*B gene sequences. A total of 76 *Micromonospora* type strains and 10 non-type strains have been used for the analysis. Distances were calculated with the Kimura 2-parameter, using 999 nucleotide positions and a bootstrap of 1,000. Bar, 0.05 substitutions per nucleotide.

The phylogenetic tree constructed with the *gyrB* gene sequences showed a similar topology to the 16S rRNA gene tree with respect to the study strains (Figure 2). These were recovered in their respective groups defined in the 16S rRNA gene tree. The exception was strain Lupac 07, recovered in the *M. noduli* cluster (Group II) and this rearrangement was supported by a bootstrap value of 99%. This strain was originally classified as *M. saelicesensis* (Trujillo et al., 2007). The positions of *M. profundi* DS 3010^T^ and *M. ureilytica* GUI23^T^ also changed and moved out of the *M. noduli* cluster. As previously noted (Garcia et al., 2010; Carro et al., 2012), the *gyrB* gene phylogeny yielded a better resolution as observed by slightly larger distance branches, however, the topology of the remaining type strains was very different from that obtained using the 16S rRNA gene. This phylogeny was very similar to a tree constructed using a concatenated set of five housekeeping genes as proposed previously (Carro et al., 2012) (data not shown).

### Comparative genomic characteristics

Eleven high quality draft genomes (depth >100X), including that of the type strain *M. noduli* GUI43^T^ were obtained. General genome characteristics of the sequenced strains are provided in Table 2. Genome sizes varied from 6.8 to 7.4 Mb, the largest genome being that of strain PSN13. The G+C mol% among all strains was very homogeneous. The values of the *M. saelicesensis* group ranged from 71.1 to 71.2% while the strains in the *M. noduli* cluster, including Lupac 07, varied from 70.9 to 71.1%. As observed, the G+C mol% values between the two species was less than 1%.

**Table 2:** General genomic characteristics of M*. saelicesensis* and *M. noduli* strains.

**Strain**	**Genome size (Mb)**	**G+C ratio (mol%)**	**Number of Contigs**	**CDS**	**rRNA operon**	**tRNA**	Genes in COGs (%)^*^	Genes in COGs (%)^**^	**Confirmed CRISPR count**
*M. saelicesensis* DSM 44871^T^	7.1	71.1	11	6568	9	67	87.13	86.13	4
GAR 05	7.0	71.2	75	6526	6	68	87.07	86.07	0
GAR 06	6.9	71.2	60	6410	8	67	89.58	88.63	4
Lupac 06	7.0	71.2	59	6495	4	65	87.62	86.61	1
PSN 01	6.8	71.1	456	6458	4	67	85.71	84.75	4
PSN 13	7.4	71.1	41	6823	3	64	86.91	85.90	1
*M. noduli* GUI43^T^	7.2	70.9	224	6539	3	57	87.81	86.88	0
LAH 08	7.2	71.1	62	6627	4	56	88.13	87.14	3
Lupac 07	7.1	71.1	56	6500	4	51	86.95	86.06	2
MED 15	7.2	71.1	43	6554	4	56	87.97	87.00	1
ONO 23	7.2	71.0	135	6565	5	56	87.39	86.43	2
ONO 86	7.1	70.9	407	6591	4	55	85.48	84.60	2

* *mapped against **bact** HMM database included in eggNOG 4.5.1*.

** *mapped against **actNOG** HMM database included in eggNOG 4.5.1*.

The number of coding DNA sequences (CDS) was also very similar between both species representatives, strain GAR06 showed the lowest number with 6410 and strain PSN13 had the highest count with 6823, a difference of 413 CDS. A slightly larger difference was observed between the number of CDS in the group of *M. saelicesensis* (410) with respect to the *M. noduli* group (127). The number of rRNA operons was also higher in *M. saelicesensis*, with the type strain Lupac 09^T^ accounting for the highest number (9 operons), followed by GAR06 (8 operons) and GAR05 (6 operons). The number of rRNA operons in the *M. noduli* was lower (3–5 operons) with the type strain, GUI43^T^ having only 3 and strain ONO23 accounting for 5. In the case of tRNAs, it was observed that the number of these molecules was higher in all strains identified as *M. saelicesensis* (64–68) than in the *M. noduli* strains (51–57). A high number of tRNAs (77) was also reported for *Micromonospora lupini* Lupac 08^T^ also isolated from nitrogen fixing nodules (Trujillo et al., 2014), while tRNAs reported for available *Micromonospora* genomes ranged from 48 to 87 (Carro et al., 2018).

The core genome of the six strains identified as *M. saelicesensis* (Group I) was calculated to be 5313 genes (81.3%) considering an average genome of 6531 genes. The number of singletons ranged from 94 for GAR06 to 706 for PSN13. In the case of the *M. noduli* strains (group II), the core genome included 5759 genes (88.05%) for an average genome of 6540 genes. In this group, strain Lupac 07 had the lowest number of singletons, 84, while strain ONO86 showed the largest variation with 369 genes. The calculation of a core genome based on all strains dropped to 74.72% and contained 4884 genes (Table S2). The calculated pangenomes were 8405, 7857, and 9867 genes for *M. saelicesensis* (Group I), *M. noduli* (Group II) and the combination of both species, respectively (Figure S1). As expected, an increase in the number of genes in the global pangenome was observed when all strains were combined, suggesting an important degree of variation between the genomes. The progression of the pan- and core genome can be seen in Figure S2.

**Figure 3:**
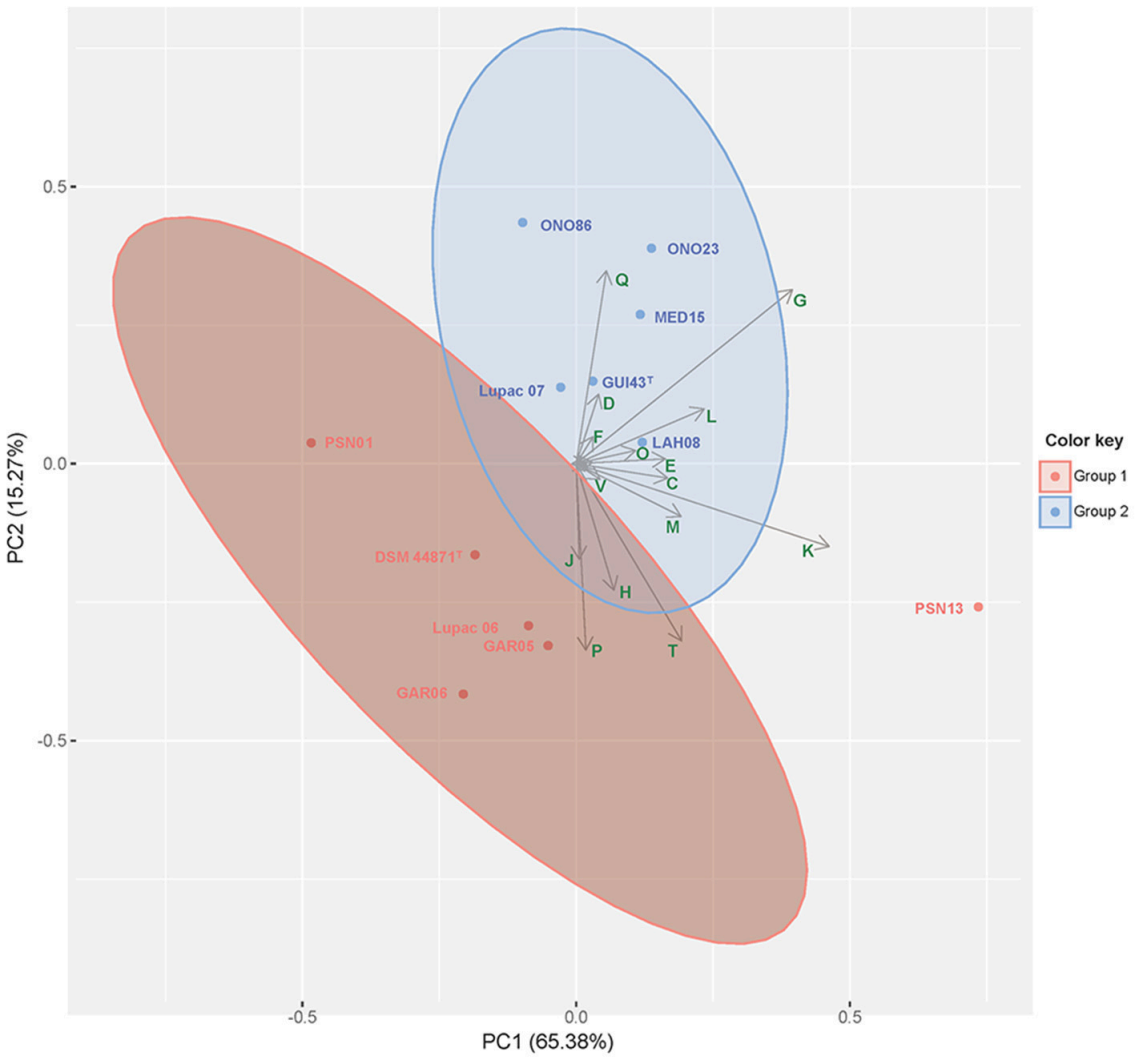
Principal Component Analysis (PCA) of COGs as distributed in the genomes of the strains representing *M. saelicesensis* (Group I) and *M. noduli* (Group II). Clustering was inferred using the K-means clustering algorithm. Arrows represent the contribution of functional COG categories. COGs represented (C) Energy production and conversion, (D) Cell cycle control, cell division and chromosome partitioning, (E) Amino acid transport and metabolism, (F) Nucleotide transport and metabolism, (G) Carbohydrate transport and metabolism, (H) Coenzyme transport and metabolism, (J) Translation, ribosomal structure and biogenesis, (K) Transcription, (L) Replication, recombination and repair, (M) Cell wall/membrane/envelope biogenesis, (O) Post-translational modification, protein turnover and chaperones, (P) Inorganic ion transport and metabolism, (Q) Secondary metabolites biosynthesis, transport and catabolism, (T) Signal transduction mechanisms, (V) Defense mechanisms. The first two principal components accounting for 80.65% of the total variance are presented in the plot.

Over 85% of the CDS for each species group were classified into Clusters of Orthologous Groups (COGs). COG profiles were very similar in all strains and were assigned into 22 categories being K (transcription, 8.6–8.9%), G (carbohydrate metabolism, 6.3–6.7%) and E (amino acid and transport metabolism, 4.7–5.0%), the most abundant. This COG distribution was very similar to the COG profile of *M. lupini* Lupac 08 (Carro et al., 2018) a close phylogenetic neighbor of *M. saelicesensis* and *M. noduli*. Principal component analysis of the COG distribution is represented in Figure 3 where both species groups are clearly separated, but with strain PNS13 recovered as an outlier. The categories K and G accounted for 65.38 and 15.27% of the variance, respectively.

### OGRI indices

Overall genomic relatedness indices (Chun and Rainey, 2014) were used to determine the relatedness between each pair of genomes used in the present study. In *M. saelicesensis* (Group I) ANI and OrthoANI values ranged from 97.82 to 99.13% and 97.96 to 99.19%, respectively, between the type and study strains. The *M. noduli* group (Group II) had ANI and OrthoANI values from 99.05 to 99.09% and 99.12 to 99.14% respectively (Table 3). In both cases, these values were above the recommended cut-off value of ~96% for species recognition (Richter and Rosselló-Móra, 2009).

**Table 3:** OGRI indices: ANI, OrthoANI, and dDDH percentage values calculated between the type and study strains.

**Strain**	**ANI *M. saelicesensis* DSM 44871^T^*/M. noduli* GUI43^T^**	**OrthoANI *M. saelicesensis* DSM 44871^T^*/M. noduli* GUI43^T^**	**dDDH *M. saelicesensis* DSM 44871^T^*/M. noduli* GUI43^T^**
*M. saelicesensis* DSM 44871^T^	100/96.64	100/96.82	100/71.20
GAR 05	99.11/96.68	99.16/96.82	92.50/71.50
GAR 06	99.09/96.61	99.18/96.79	92.90/71.30
Lupac 06	99.13/96.63	99.19/96.82	92.70/71.20
PSN 01	99.12/96.61	99.19/96.82	92.80/71.60
PSN 13	97.82/96.55	97.96/96.79	81.30/71.30
	***M. noduli*** **GUI43**^T^***/M. saelicesensis DSM*** **44871**^T^	***M. noduli*** **GUI43**^T^***/M. saelicesensis DSM*** **44871**^T^	***M. noduli*** **GUI43**^T^***/M. saelicesensis DSM*** **44871**^T^
*M. noduli* GUI43^T^	100/96.64	100/96.82	100/71.20
LAH 08	99.09/96.23	99.15/96.83	92.50/71.30
Lupac 07	99.09/96.59	99.14/96.80	92.70/71.30
MED 15	99.09/96.59	99.13/96.78	92.60/71.10
ONO 23	99.05/96.62	99.12/96.87	92.30/71.30
ONO 86	99.05/96.59	99.14/96.23	92.80/71.50

The ANI and OrthoANI values between the type strains *M. saelicesensis* and *M. noduli* were 96.64 and 96.82 respectively. Overall, pairwise comparison between the two groups showed the highest ANI and OrthoANI values corresponded to strains GAR05 and GUI43^T^ (96.68%, ANI) and PSN01 and ONO23 (96.90%, OrthoANI) (Table S3). Both results are slightly above the border line of 95–96% for the delineation of species. However, these results are comparable to OrthoANI values obtained for the genome pairs of *M. carbonacea* and *M. haikouensis* (95.16%), and *M. inyonensis* and *M. sagamiensis* (96.5%).

**Figure 4:**
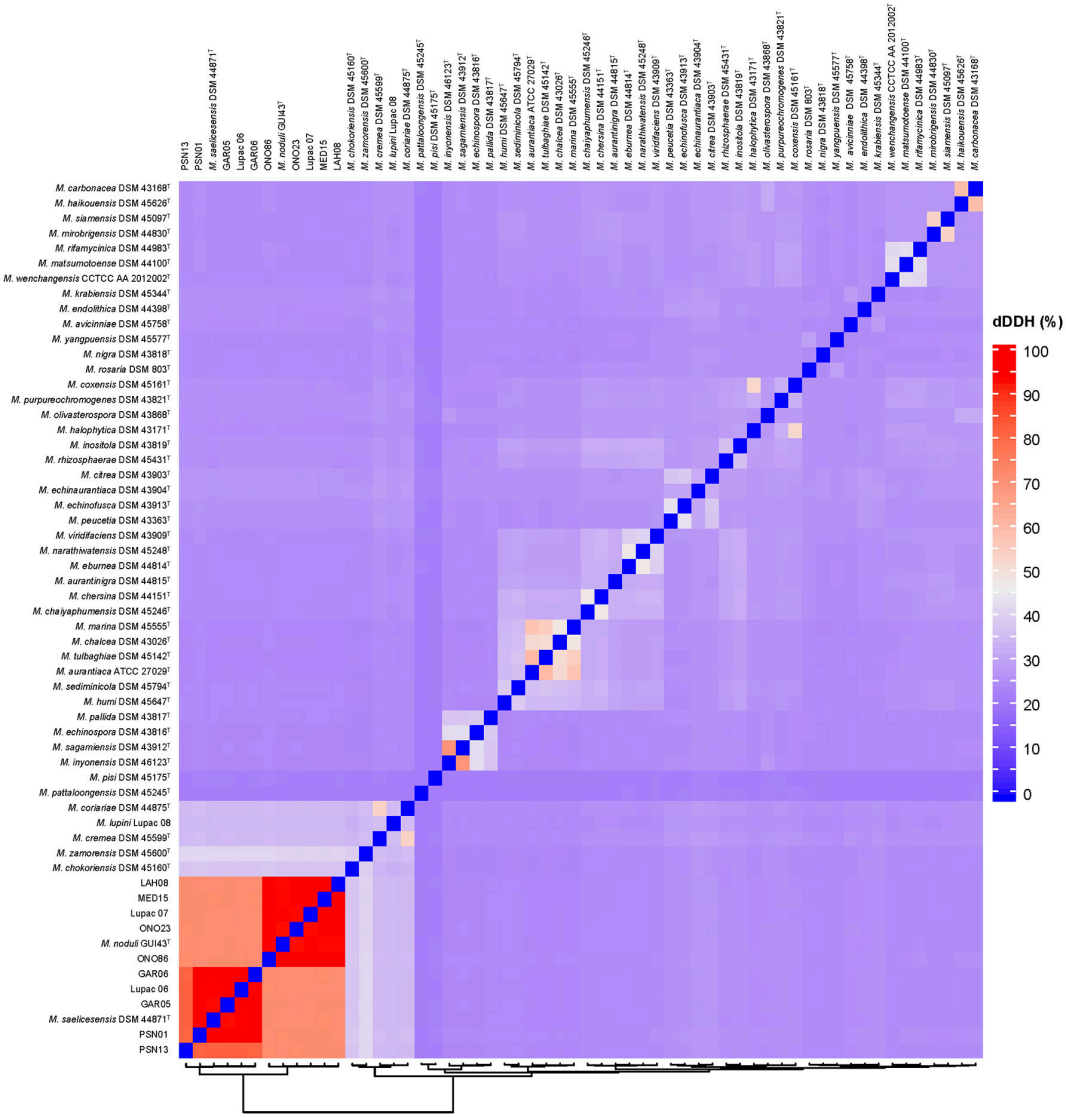
Digital DNA-DNA hybridization (dDDH) pairwise comparison heatmap. The two red squares correspond to the delineation of the species *Micromonospora saelicesensis* and *M. lupini* with values ranging from 81 to 93.7%. Interspecies limits are between 71.0 and 71.8%.

Species delineation based on dDDH values ranged from 81.0 to 93.7% for the six strains in *M. saelicesensis* (Group I) and 92.3–93.8% for the 5 components of *M. noduli* (Group II); all values clearly above the 70% recommended threshold (Table S3). dDDH values between the two species groups ranged from 71.0 to 71.8% (Table 3). Similar to ANI and OrthoANI results, dDDH values between the two groups were slightly above the border limit threshold value of 70% (68.1–74.5%). Overall pairwise comparisons of the study genomes and 48 additional *Micromonospora* type strains show the close relationship of the strains but clearly delineate each group within this 70–71% dDDH radius (Figure 4). A similar situation is observed between the species *Micromonospora sagamiensis* and *Micromonospora inyonensis* which share a dDDH value close to 70% (69.8%, dDDH; 61.3%, experimental DDH) (Kroppenstedt et al., 2005). Meier-Kolthoff and colleagues (Meier-Kolthoff et al., 2014), recently proposed the delineation of subspecies using genomic data. Specifically, these authors recommend a threshold of 79–80% to define subspecies in prokaryotic taxonomy. In the present study, the values obtained for the *Micromonospora* strains are much lower than this range and these strains are better classified as different species rather than subspecies.

### Whole-genome phylogenomic analysis

**Figure 5:**
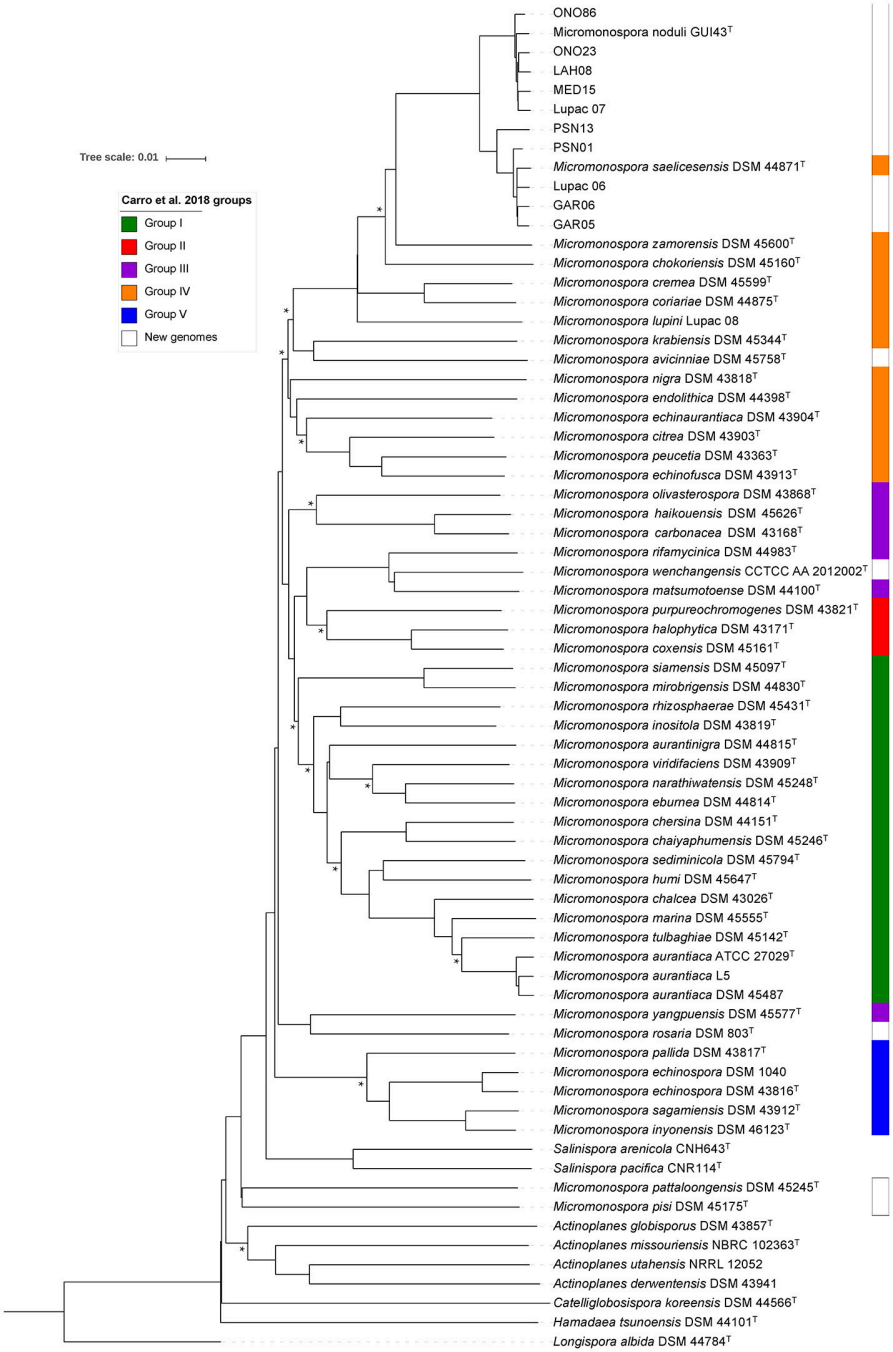
Whole genome-sequence based phylogenomic tree constructed with the GBDP tool (see main text for details). Colors on the right side represent groups described in Carro et al. (2018). Asterisks represent conserved nodes between this tree and the core genome phylogenetic tree.

Phylogenomic tree reconstruction based on whole-genome distances calculated with the GBDP tool is presented in Figure 5. This tree included the 10 study genomes, all *Micromonospora* strains (type and non-type) published previously (Carro et al., 2018) and the genome sequences of the type strains *M. noduli* GUI43^T^ (this work), *M. avicinniae* DSM 45748^T^, *M. pisi* DSM 45175^T^*, M. pattaloongensis* DSM 45245^T^, *M. rosaria* DSM 803^T^ and *M. wenchangensis* CCTCC AA 2012002^T^. The composition of the *M. saelicesensis* and *M. noduli* groups defined in the *gyrB* gene tree were identical, including the position of Lupac 07 as a member of *M. noduli* (Group II). The overall topology of this tree and the one published by Carro et al. (2018) was very similar, however, the inclusion of 17 additional genomes, as expected, influenced the distribution of the type strains, especially the inclusion of the six additional type strains. Nevertheless, three out of Carro's five defined groups (I, IV, and V) were almost completely recovered in the present phylogenomic analysis, the major rearrangements were observed in Carro's groups II and III. In the present phylogenomic analysis, the strains in group II (*M. purpureochromogenes, M. coxensis* and *M. halophytica*) fused with *M. rifamycinica* and *M. matsumotoense* (group III) and were joined by *M. wenchangensis* (new to the analysis). The instability of group III was already highlighted (Carro et al., 2018). This rearrangement reduced group III to *M. olivasterospora, M. carbonacea*, and *M. haikouensis*.

### UBCG phylogenomic analysis

**Figure 6:**
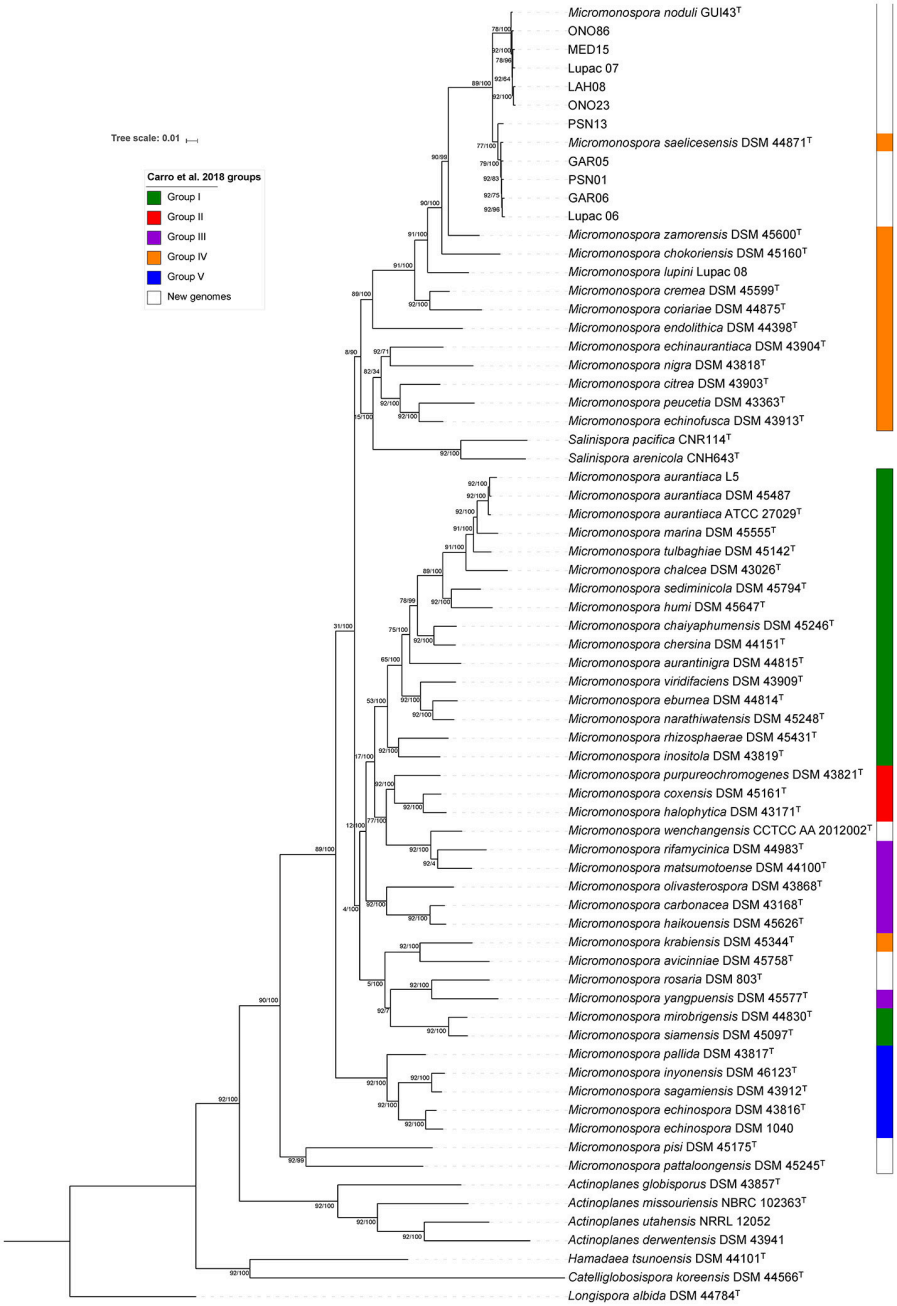
Up-to-date bacterial core gene phylogeneomic tree reconstructed with 92 bacterial core genes. Tree has been formatted using itol platform. Colors on the right represent groups described in Carro et al. (2018). GSI support (left) and bootstrap values(right) are given at nodes.

The same dataset as above was used to construct a phylogenetic tree based on a core genome set of 92 genes using the UBCG tool (Na et al., 2018). Most of the selected genes (67/92) fall in the translation COG category (J), coding for ribosomal proteins (25/92, 50S and 18/92, 30S), aminoacid-tRNA ligases (10/92) and elongation an initiator factors (4/92) (Table S4). Again, the ten strains were distributed in two groups of identical composition as that of the *gyrB* gene and whole-genome phylogenomic analyses with significant branch support as indicated by the bootstrap values and gene support indices (GSI) (Figure 6). GSI values indicate the reliability of the branches on the phylogenomic tree based on the total number of genes used to construct the tree (92 genes) (Na et al., 2018). The topology of this tree with respect to the composition of the two groups was the same as the whole-genome and *gyrB* gene trees, including the position of strain Lupac 07, recovered in the *M. noduli* group. The topology of the UBCG tree highly correlated to the topology of the whole-genome phylogenomic tree of this study. Especially interesting was the fact that the new redefined groups II and III were recovered in their entirety together with groups I, IV and V. In this analysis, a new group that contained strains from Carro's groups I (*M. mirobrigensis* and *M. siamensis*), III (*M. yangpuensis*) and IV (*M. krabiensis*), in addition to the newly included type strains *M. avicinniae* and *M. rosaria* was formed (Figure 6). Another important difference between the whole- and UBCG trees of this study was the position of *Salinispora pacifica* and *Salinispora arenicola* which in the latter tree was found associated to group IV. In this case, the up-to-date bacterial core gene analysis was not resolutive.

### Phenotypic profiles

Thirty-one phenotypic tests reported previously to be useful for the differentiation of the species *M. saelicesensis* and *M. noduli* (Carro et al., 2016) were carried out with all test strains. The number of characteristics that phenotypically differentiated between the two species was significantly reduced to one test when the number of strains compared increased (Table S5). Specifically, the use of rhamnose as a carbon source substrate was positive for all strains in the *M. noduli* group while the results were all negative for *M. saelicesensis* strains except for isolate PSN13 which was positive. The results of the remaining tests varied at the strain level and did not relate to their species identification.

Intra-species variability within each species group ranged from 0 to 33.3%. Range of pH growth and lipase production were the most variable tests in *M. saelicesensis*; utilization of serine as carbon source, degradation of tyrosine and pH growth range were the most variable tests for the *M. noduli* group.

Phenotypic profiles using the Biolog system were also determined for all strains. In this case, none of the 71 carbon sources or the 23 biochemical tests served to differentiate between the two species, given the variability observed among the duplicate tests (Table S6). Strain Lupac 06 was the most variable with 35.1% discrepancies recorded. Overall intraspecies variability for *M. saelicesensis* and *M noduli* was 25.5 and 26.6% respectively.

Nineteen carbon sources were also assayed at different times (2007, 2016, and 2017) to check for reproducibility. Nine of the eleven strains tested expressed discrepant results over the different testing times. Three strains (Lupac 09^T^, Lupac 06, and Lupac 07) showed the highest variation with 26% of the tests yielding conflicting results while MED15, LAH08, and PSN13 had the lowest variation (5.2%). The use of D-serine as carbon source was the least reproducible test with seven strains yielding conflicting results (Table S7).

Draft genomes of the test strains were screened for genes involved in the carbon metabolism of the corresponding 19 substrates assayed *in vitro*. The predicted phenotypes correlated 100% with the results obtained in the laboratory for 11 tests. However, in the case of L-alanine, L-arginine, L-histidine, L-lysine, myo-inositol, L-rhamnose, D-serine, and D-trehalose, discrepant results were found between wet lab and *in silico* predictions (Figure S3). In most cases, the genes were localized in the genome but the experimental results varied (+/–) suggesting that even when the tests were carried out in the same laboratory and using the same method, they were not 100% reproducible.

In the case of L-rhamnose, *in vitro* tests for strain GUI43^T^ were positive but the genes related to the metabolism of this compound were not located. This is probably explained by the fact that draft-genomes were used and interpretation of genomic data should be done with precaution.

## Discussion

The genus *Micromonospora* is highly relevant in biotechnological applications in areas such as medicine, agriculture and biofuels (Hirsch and Valdés, 2010; Trujillo et al., 2015; Carro et al., 2018). At present, this taxon holds 81 species with validly published names (LPSN), most of them described based on a polyphasic approach (Colwell, 1970; Vandamme et al., 1996). Within this framework, DNA-DNA hybridization (DDH) has been considered the key test to decide if a new strain represents a new species, despite its well spelled limitations (Gevers et al., 2005; Meier-Kolthoff et al., 2013a). Given the drawbacks of DDH, it is not always straight forward to delineate the species limits, especially when DDH values close to the threshold. Therefore, the development of whole genome sequencing seems more appropriate to deduce relatedness by comparing genome sequences rather than performing DDH experiments (Vandamme and Peeters, 2014). Genomic data was recently used as the backbone to revisit the classification of the genus *Micromonospora* using a set of 45 draft genomes providing a useful dataset for comparison (Carro et al., 2018).

While 16S rRNA is limited in resolving phylogenetic relationships at the species level (Katayama et al., 2007; Hahnke et al., 2016; Carro et al., 2018; Na et al., 2018), it has provided a good starting point for taxonomic studies. In this work, 16S rRNA gene sequencing was used to identify the closest neighbors of ten *Micromonospora* strains isolated from various legumes (Table 1). The sequence similarity values indicated that *M. saelicesensis* or *M. noduli* were the two most closely related species although in some cases, similarity values were identical between the test and both type strains (e.g., GAR06 and LAH08). The 16S rRNA gene tree topology yielded two very tight groups which could be interpreted as a single one when the branch lengths from these clusters were compared against the lengths of the remaining 79 *Micromonospora* type strains included in the analysis.

The use of *gyrB* gene sequences to resolve phylogenetic relationships in the genus *Micromonospora* has been recommended by several authors (Kasai et al., 2000; Garcia et al., 2010; Carro et al., 2012) given its higher resolution when compared to 16S rRNA gene phylogeny. In this study, the *gyrB* gene tree topology showed a similar arrangement to the 16S rRNA gene tree with respect to the test strains, however several differences were observed. The branch lengths were slightly longer, but still very small when compared to the rest of the *Micromonospora* species included in the tree. The most relevant change was the position of strain Lupac 07, which, together with strains Lupac 06 and Lupac 09^T^ were originally classified as *M. saelicesensis* (Trujillo et al., 2007). The latter strains remained in the *M. saelicesensis* cluster but Lupac 07 moved to the *M. noduli* group. As expected, topologies of both trees in relation to the type strains were very different confirming that phylogenies based on single genes are very limited and unstable, making identification of nearest phylogenetic neighbors difficult.

The tree topologies based on the phylogenomic analyses of the UBCG (92 genes) and the whole draft genomes were similar. In both trees, strain Lupac 07 was recovered in the *M. noduli* group, strongly suggesting that this strain should be reclassified as a member of this species. The remaining 9 strains were recovered in the same species groups throughout all analyses.

In this study, both phylogenomic analyses contained a total of 70 genomes, including six additional *Micromonospora* type strains (see above). Overall, good agreement was found between the two phylogenies of this work and recently published data. In all cases, groups I, IV, and V previously defined (Carro et al., 2018) were recovered in their entirety with *M. avinniceae* (this analysis) joining group IV. The main difference between the three phylogenies was the composition of Carro's groups II and III which were clearly influenced by the addition of *M. rosaria* DSM 803^T^ and *M. wenchangensis* CCTCC AA 2012002^T^, producing a new group recovered in both phylogenies of the present work. Nevertheless, the groups I, IV, and V remained very stable considering that 11 new genomes (*M. noduli* GUI43^T^ and 10 test strains) were added and these were assigned to group IV where *M. saelicesensis* Lupac 09 was originally assigned. These rearrangements reinforce the argument that classification and identification systems are data dependent and constant rearrangement should be expected as more data are added and alternative methods are applied (Carro et al., 2018).

The new analysis tool UBCG proved useful for the construction of phylogenomic analysis, showing good correlation with trees using whole-draft genome data even though it did not resolve well the position of the *Salinispora* representatives, however, this may be due to the small number of representatives in the data set. An advantage of this pipeline is the use of bootstrap and GSI values to support the phylogenetic branches. It is also expected that as more genome sequences are added to the database, the more resolutive it should become.

Genome relatedness indices (ANI, Ortho-ANI, and dDDH) were calculated to complement the phylogenomic analyses for species demarcation. Overall, the three methods showed good agreement and the two species groups defined in the *gyrB*, core-genome and whole-genome phylogenetic analyses supported the recognition of the 10 strains in two species.

Furthermore, these studies served to highlight the close relationship between the species *M. saelicesensis* and *M. noduli*. ANI values proposed for species delineation have been set to 95–96% as this range has been found to be correlated with the experimental DDH threshold of 70% (Goris et al., 2007; Richter and Rosselló-Móra, 2009). An alternative means to measure relatedness between two genomes is the calculation of dDDH using the GBDP method which appears to show a better correlation than ANI to the data derived from DDH experiments (Auch et al., 2010; Meier-Kolthoff et al., 2013b; Peeters et al., 2016).

In this work, the OGRI values were slightly above the recommended threshold for species delineation, if strictly applied, the study strains should be recognized as members of the same species. However, the consideration of other results in this work support the recognition of the strains as two separate species, *M. saelicesensis* and *M. noduli*. As previously expressed, thresholds are necessary for guidance but these should be applied in a flexible manner and considering other biological properties (Li et al., 2015). The present work is a good example for the interpretation and application of these values.

The use of phenotypic traits to identify and differentiate species in prokaryotic systematics is of limited value as previously discussed (Sutcliffe et al., 2013; Amaral et al., 2014; Vandamme and Peeters, 2014). In this work, several strains identified as one species, expressed different phenotypes, highlighting the problem of using diagnostic tables based on single strains to list differential characteristics between species. Information about intra-species variation is crucial for the development of stable diagnostic characteristics and the convenience of using more than a single isolate have been previously discussed (Sutcliffe et al., 2012; Oren and Garrity, 2014).

Our results confirm that the use of phenotypic tests, even when performed under the same conditions are not reliable for species differentiation due to the high variability observed within several members of the same species (Kumar et al., 2015). Instead, phenotypic studies should be regarded as complementary information to understand the biology of a microorganism and they should be restricted to strain characterization. Understandably, the inclusion of additional strains for the description of a taxon is often regarded as a burden because a lot of extra work is needed, especially when looking for differential phenotypic tests with questionable taxonomic value (Sutcliffe et al., 2012; Vandamme and Peeters, 2014).

Genomic information can be used to determine the intrinsic variability between a set of strains based on the core and pangenome profiles (Coenye et al., 2005; Sutcliffe et al., 2012; Oren and Garrity, 2014). In this work the calculation of these parameters has pointed out an important degree of variation between the species *M. saelicesensis* and *M. noduli* supporting their recognition as separate taxa. The complete elucidation of the gene functions within each group may provide an initial set of stable differential characteristics for each species, some of which may be phenotypically expressed.

## Concluding remarks

As additional data is generated, genome-based classifications should become more stable and provide a new working frame for the systematics of prokaryotes. The present study illustrates the advantage of using a diverse array of methods for the correct identification of new strains and the importance of using more than one isolate for a better characterization and definition of a species. OGRI values and especially dDDH values seem very appropriate for the delineation of prokaryotic species, but threshold numbers should be applied with a sufficient level of flexibility and considering other features inherent to a microorganism such as ecology, physiology, etc. (Li et al., 2015). There is no doubt that phenotypic information is useful for the good characterization of strains, but these studies should aim to provide information on the biology of a microorganism and not necessarily and not only to fill out a table with results of questionable value.

## Author contributions

RR, LC and BR-P carried out experiments and bioinformatic analyses. CP and JB performed bioinformatic analyses. H-PK, PN, and MT designed the study and wrote the manuscript. All authors read the manuscript.

### Conflict of interest statement

The authors declare that the research was conducted in the absence of any commercial or financial relationships that could be construed as a potential conflict of interest.

## Supplementary material

Figure S1 **(A)** Venn diagram showing the number of orthologous genes clusters that integrate the core and disposable genomes, and singletons of all strains in Group I (*Micromonospora saelicesensis*). **(B)** Venn diagram showing the number of orthologous gene clusters that integrate the core genome and disposable genomes, and singletons of all strains in Group II (*Micromonospora noduli*).

Figure S2 Pan- and Core genome development plot of *Micromonospora noduli* and *Micromonospora saelicesensis* strains. The orange and blue lines show the progression in the pan- and core genomes as more genomes are added.

Figure S3 Predicted phenotypes vs. experimental phenotypic data based on 19 carbon source substrates. *In silico* prediction negative, phenotype not expressed (purple); *in silico* prediction negative (genes not found), phenotype expressed (red); *in silico* prediction positive, phenotype not expressed (light green) and *in silico* prediction positive, phenotype expressed (green).

Table S1 Genome sequence accession numbers of strains used in this work.

Table S2 Number of orthologous genes that conform the pan genome, core genome and singletons of *Micromonospora saelicesensis* (Group 1) and *Micromonospora noduli* (Group II). In parenthesis, values expressed as percentages based on an average genome of 6531 genes for *M. saelicesensis* and 6540 genes for *M. noduli*.

Table S3 Pair-wise OGRI values for ANI, OrthoANI and dDDH between *M. saelicesensis* (Group I) and *M. noduli* (Group II) strains.

Table S4 List of genes used in core-genome phylogenomic analysis based on UBCG (Na et al., 2018).

Table S5 Differential phenotypic characteristics between *M. saelicesensis* and *M. noduli* as reported by Carro and colleagues (Carro et al., 2016). +, Positive; –, Negative; w, Weak.

Table S6 BIOLOG phenotypic profiles of *M. saelicesensis* and *M. noduli* strains. +, positive; –, negative; c, conflicting.

Table S7 Carbon source substrates tested at different times using the same laboratory conditions. +, positive; –, negative; w, weak.
