# Supplementary figures and images for: Defining the Species Micromonospora saelicesensis and Micromonospora noduli Under the Framework of Genomics
Source: Front Microbiol. 2018 Jun 25;9:1360. doi: 10.3389/fmicb.2018.01360 (PMC6026663; doi:10.3389/fmicb.2018.01360)

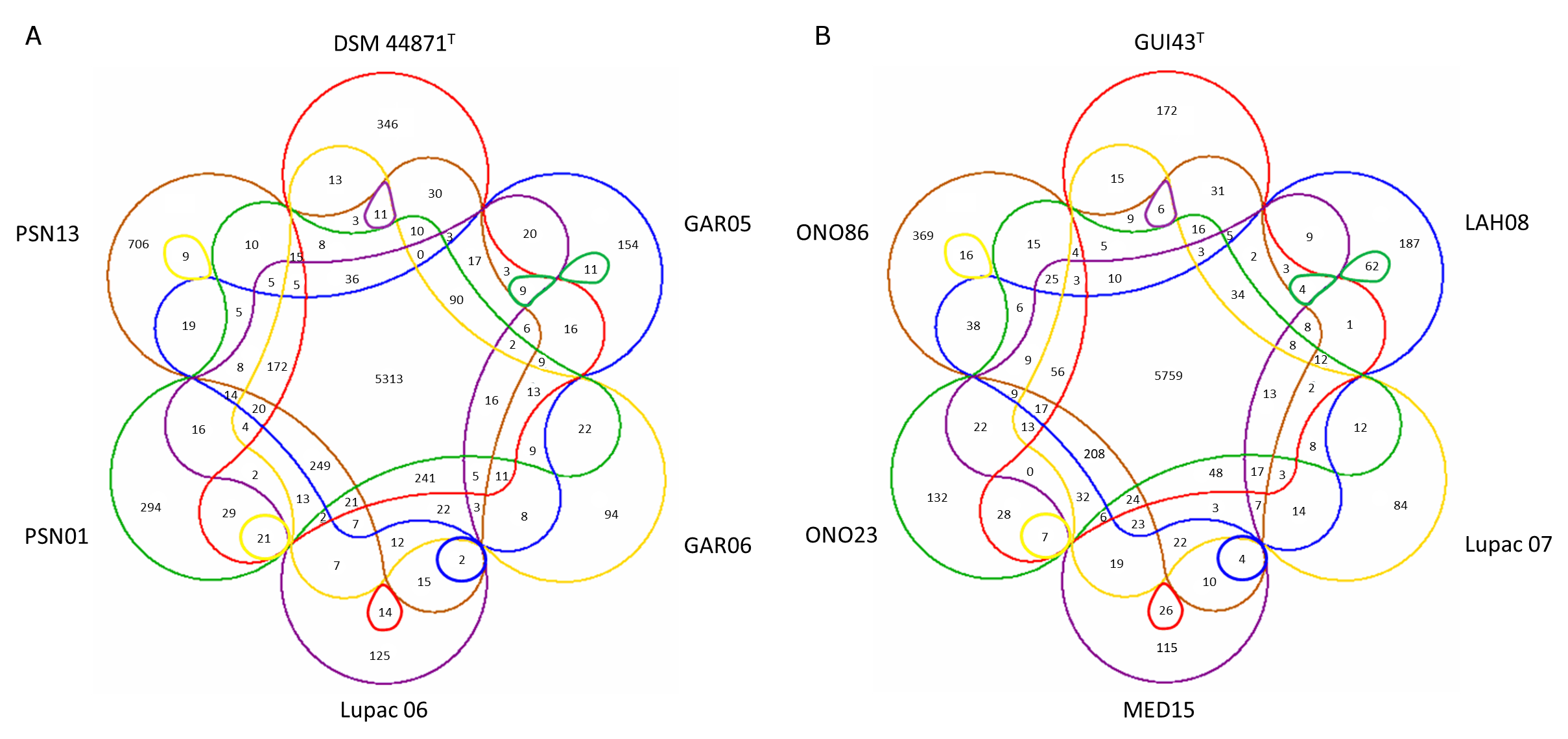

Supplement: Figure S1 — (A) Venn diagram showing the number of orthologous genes clusters that integrate the core and disposable genomes, and singletons of all strains in Group I (Micromonospora saelicesensis). (B) Venn diagram showing the number of orthologous gene clusters that integrate the core genome and disposable genomes, and singletons of all strains in Group II (Micromonospora noduli). [file Image_1.TIFF]

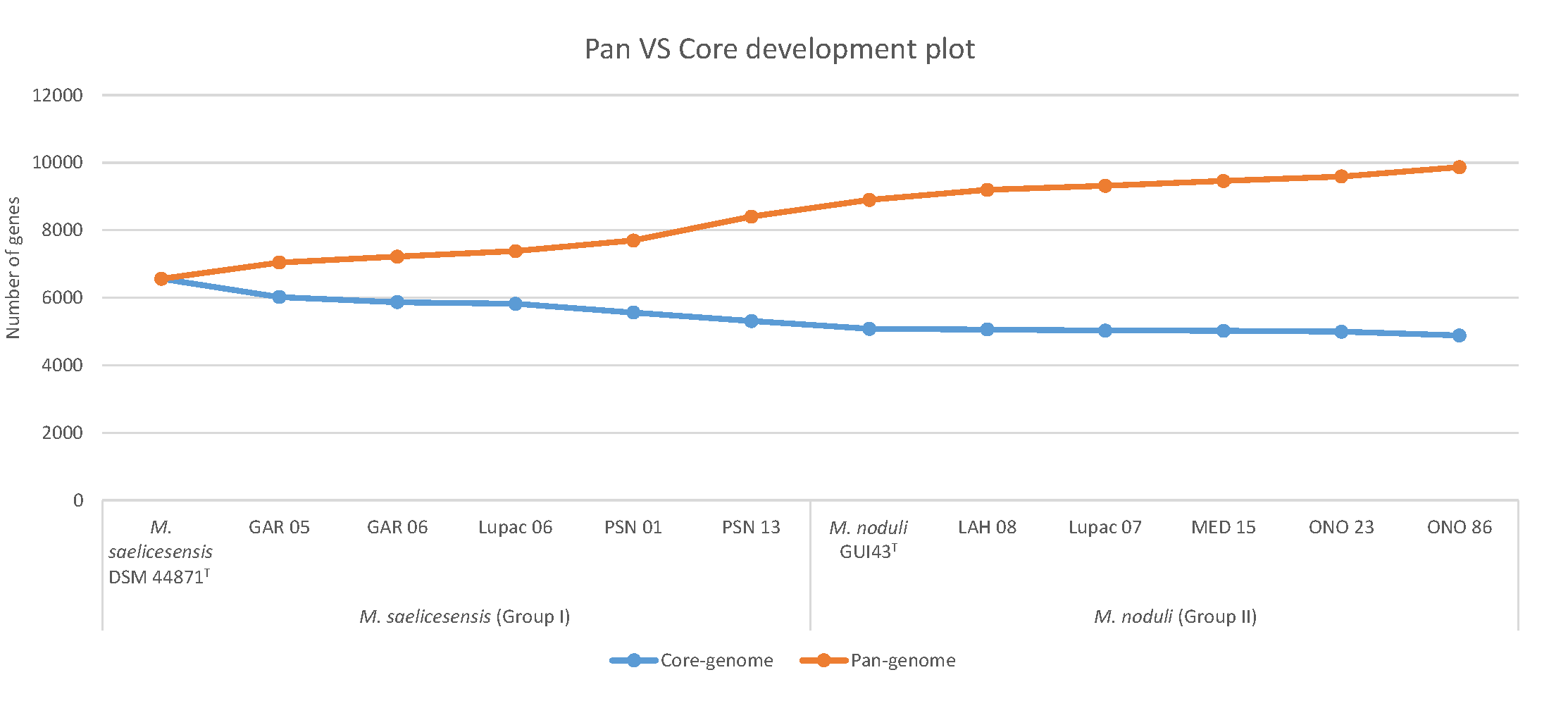

Supplement: Figure S2 — Pan- and Core genome development plot of Micromonospora noduli and Micromonospora saelicesensis strains. The orange and blue lines show the progression in the pan- and core genomes as more genomes are added. [file Image_2.TIFF]

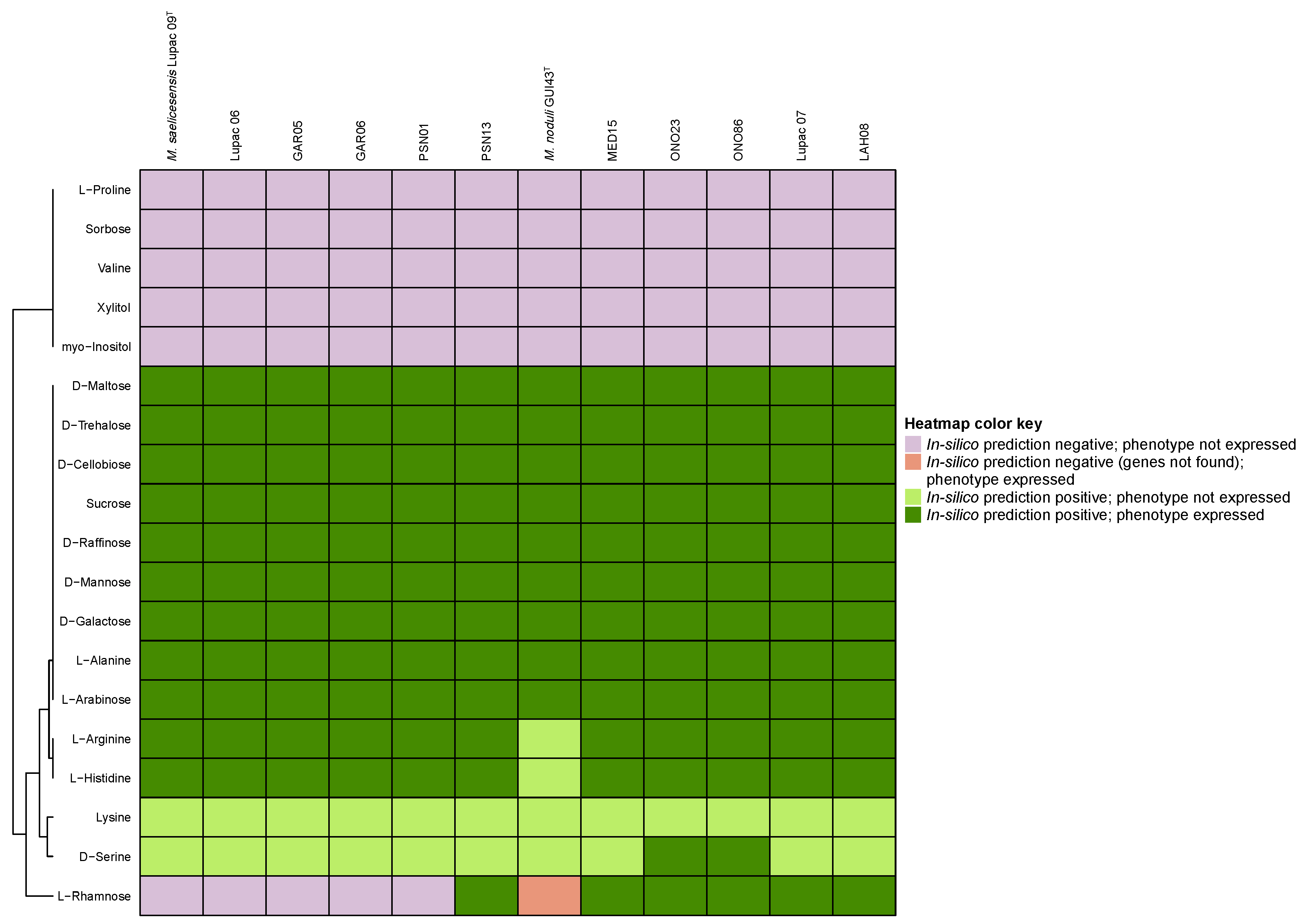

Supplement: Figure S3 — Predicted phenotypes vs. experimental phenotypic data based on 19 carbon source substrates. In silico prediction negative, phenotype not expressed (purple); in silico prediction negative (genes not found), phenotype expressed (red); in silico prediction positive, phenotype not expressed (light green) and in silico prediction positive, phenotype expressed (green). [file Image_3.TIFF]
