# Supplementary material for: Defining the Species Micromonospora saelicesensis and Micromonospora noduli Under the Framework of Genomics
Source: Front Microbiol. 2018 Jun 25;9:1360. doi: 10.3389/fmicb.2018.01360 (PMC6026663; doi:10.3389/fmicb.2018.01360)
Supplement: Table S2 — Number of orthologous genes that conform the pan genome, core genome and singletons of Micromonospora saelicesensis (Group 1) and Micromonospora noduli (Group II). In parenthesis, values expressed as percentages based on an average genome of 6531 genes for M. saelicesensis and 6540 genes for M. noduli. [file Table_2.DOCX]

**Table S2.** Number of orthologous genes that conform the pan genome, core genome and singletons of *Micromonospora saelicesensis* (Group 1) and *Micromonospora noduli* (Group II). In parenthesis, values expressed as percentages based on an average genome of 6531 genes for *M. saelicesensis* and 6540 genes for *M. noduli.*

|  | Pan genome | Core genome | Singletons | |
| --- | --- | --- | --- | --- |
|  |  |  | Strain | Gene count (%) |
| *M. saelicesensis* (Group I) | 8405 | 5313 (81.35%) | DSM 44871^T^ | 346 (5.29%) |
|  |  |  | GAR05 | 154 (2.36%) |
|  |  |  | GAR06 | 94 (1.44%)) |
|  |  |  | Lupac 06 | 125 (1.91%) |
|  |  |  | PSN01 | 294 (4.50%) |
|  |  |  | PSN13 | 706 (10.08%) |
| *M. noduli*  (Group II) | 7857 | 5759 (88.05%) | GUI43^T^ | 172 (2.63%) |
|  |  |  | LAH08 | 187 (2.86%) |
|  |  |  | Lupac 07 | 84 (1.28%) |
|  |  |  | MED15 | 115 (1.76%) |
|  |  |  | ONO23 | 132 (2.02%) |
|  |  |  | ONO86 | 369 (5.64%) |
