# Supplementary material for: Defining the Species Micromonospora saelicesensis and Micromonospora noduli Under the Framework of Genomics
Source: Front Microbiol. 2018 Jun 25;9:1360. doi: 10.3389/fmicb.2018.01360 (PMC6026663; doi:10.3389/fmicb.2018.01360)
Supplement: Table S4 — List of genes used in core-genome phylogenomic analysis based on UBCG (Na et al., 2018). [file Table_4.DOCX]

**Table S4**. List f genes used in core-genome phylogenomic analysis based on UBCG (Na et al. 2018).

| **Gene** | **COG category** | **COG ID** | **HMM profile** | **Function** |
| --- | --- | --- | --- | --- |
| *alaS* | J | COG0013 | TIGR00344 | Alanine-tRNA ligase |
| *argS* | J | COG0018 | TIGR00456 | Arginine-tRNA ligase |
| *aspS* | J | COG0173 | TIGR00459 | Aspartate-tRNA ligase |
| *cgtA* | DL | COG0536 | TIGR02729 | GTPase ObgE/CgtA |
| *coaE* | H | COG0237 | TIGR00152 | Dephospho-CoA kinase |
| *cysS* | J | COG0215 | TIGR00435 | Cysteine-tRNA ligase |
| *dnaA* | L | COG0593 | TIGR00362 | Chromosomal replication initiator protein DnaA |
| *dnaG* | L | COG0358 | TIGR01391 | DNA primase |
| *dnaX* | L | COG2812 | TIGR02397 | DNA polymerase III subunit gamma |
| *engA* | R | COG1160 | TIGR03594 | GTPase Der |
| *ffh* | U | COG0541 | TIGR00959 | Signal recognition particle protein |
| *fmt* | J | COG0223 | TIGR00460 | Methionyl-tRNA formyltransferase |
| *frr* | J | COG0233 | TIGR00496 | Ribosome-recycling factor |
| *ftsY* | U | COG0552 | TIGR00064 | Signal recognition particle receptor FtsY |
| *gmk* | F | COG0194 | TIGR03263 | Guanylate kinase |
| *hisS* | J | COG0124 | TIGR00442 | Histidine-tRNA ligase |
| *ileS* | J | COG0060 | TIGR00392 | Isoleucine-tRNA ligase 1 |
| *infB* | J | COG0532 | TIGR00487 | Translation initiation factor IF-2 |
| *infC* | J | COG0290 | TIGR00168 | Translation initiation factor IF-3 |
| *ksgA* | J | COG0030 | TIGR00755 | Ribosomal RNA small subunit methyltransferase A |
| *lepA* | J | COG0481 | TIGR01393 | Elongation factor 4 |
| *leuS* | J | COG0495 | TIGR00396 | Leucine-tRNA ligase |
| *ligA* | L | COG0272 | TIGR00575 | DNA ligase |
| *nusA* | K | COG0195 | TIGR01953 | Transcription termination/antitermination protein NusA |
| *nusG* | K | COG0250 | TIGR00922 | Transcription termination/antitermination protein NusG |
| *pgk* | G | COG0126 | PF00162 | Phosphoglycerate kinase |
| *pheS* | J | COG0016 | TIGR00468 | Phenylalanine-tRNA ligase alpha subunit |
| *pheT* | J | COG0073 | TIGR00472 | Phenylalanine-tRNA ligase beta subunit |
| *prfA* | J | COG0216 | TIGR00019 | Peptide chain release factor 1 |
| *pyrG* | F | COG0504 | TIGR00337 | CTP synthase |
| *rbfA* | J | COG0858 | TIGR00082 | 30S ribosome-binding factor |
| *recA* | L | COG0468 | TIGR02012 | DNA recombination and repair protein |
| *rnc* | K | COG0571 | TIGR02191 | Ribonuclease 3 |
| *rplA* | J | COG0081 | TIGR01169 | 50S ribosomal protein L1 |
| *rplB* | J | COG0090 | TIGR01171 | 50S ribosomal protein L2 |
| *rplC* | J | COG0087 | TIGR03625 | 50S ribosomal protein L3 |
| *rplD* | J | COG0088 | TIGR03953 | 50S ribosomal protein L4 |
| *rplE* | J | COG0094 | PF00281 | 50S ribosomal protein L5 |
| *rplF* | J | COG0097 | TIGR03654 | 50S ribosomal protein L6 |
| *rplI* | J | COG0359 | TIGR00158 | 50S ribosomal protein L9 |
| *rplJ* | J | COG0244 | PF00466 | 50S ribosomal protein L10 |
| *rplK* | J | COG0080 | TIGR01632 | 50S ribosomal protein L11 |
| *rplL* | J | COG0222 | TIGR00855 | 50S ribosomal protein L7/L12 |
| *rplM* | J | COG0102 | TIGR01066 | 50S ribosomal protein L13 |
| *rplN* | J | COG0093 | TIGR01067 | 50S ribosomal protein L14 |
| *rplO* | J | COG0200 | TIGR01071 | 50S ribosomal protein L15 |
| *rplP* | J | COG0197 | TIGR01164 | 50S ribosomal protein L16 |
| *rplQ* | J | COG0203 | TIGR00059 | 50S ribosomal protein L17 |
| *rplR* | J | COG0256 | TIGR00060 | 50S ribosomal protein L18 |
| *rplS* | J | COG0335 | TIGR01024 | 50S ribosomal protein L19 |
| *rplT* | J | COG0292 | TIGR01032 | 50S ribosomal protein L20 |
| *rplU* | J | COG0261 | TIGR00061 | 50S ribosomal protein L21 |
| *rplV* | J | COG0091 | TIGR01044 | 50S ribosomal protein L22 |
| *rplW* | J | COG0089 | PF00276 | 50S ribosomal protein L23 |
| *rplX* | J | COG0198 | TIGR01079 | 50S ribosomal protein L24 |
| *rpmA* | J | COG0211 | TIGR00062 | 50S ribosomal protein L27 |
| *rpmC* | J | COG0255 | TIGR00012 | 50S ribosomal protein L29 |
| *rpmI* | J | COG0291 | TIGR00001 | 50S ribosomal protein L35 |
| *rpoA* | K | COG0202 | TIGR02027 | DNA-directed RNA polymerase subunit alpha |
| *rpoB* | K | COG0085 | TIGR02013 | DNA-directed RNA polymerase subunit beta |
| *rpoC* | K | COG0086 | TIGR02386 | DNA-directed RNA polymerase subunit beta’ |
| *rpsB* | J | COG0052 | TIGR01011 | 30S ribosomal protein S2 |
| *rpsC* | J | COG0092 | TIGR01009 | 30S ribosomal protein S3 |
| *rpsD* | J | COG0522 | TIGR01017 | 30S ribosomal protein S4 |
| *rpsE* | J | COG0098 | TIGR01021 | 30S ribosomal protein S5 |
| *rpsF* | J | COG0360 | TIGR00166 | 30S ribosomal protein S6 |
| *rpsG* | J | COG0049 | TIGR01029 | 30S ribosomal protein S7 |
| *rpsH* | J | COG0096 | PF00410 | 30S ribosomal protein S8 |
| *rpsI* | J | COG0103 | PF00380 | 30S ribosomal protein S9 |
| *rpsJ* | J | COG0051 | TIGR01049 | 30S ribosomal protein S10 |
| *rpsK* | J | COG0100 | TIGR03632 | 30S ribosomal protein S11 |
| *rpsL* | J | COG0048 | TIGR00981 | 30S ribosomal protein S12 |
| *rpsM* | J | COG0099 | TIGR03631 | 30S ribosomal protein S13 |
| *rpsO* | J | COG0184 | TIGR00952 | 30S ribosomal protein S15 |
| *rpsP* | J | COG0228 | TIGR00002 | 30S ribosomal protein S16 |
| *rpsQ* | J | COG0186 | TIGR03635 | 30S ribosomal protein S17 |
| *rpsR* | J | COG0238 | TIGR00165 | 30S ribosomal protein S18 |
| *rpsS* | J | COG0185 | TIGR01050 | 30S ribosomal protein S19 |
| *rpsT* | J | COG0268 | TIGR00029 | 30S ribosomal protein S20 |
| *secA* | U | COG0653 | TIGR00963 | Protein translocase subunit SecA |
| *secG* | U | COG1314 | TIGR00810 | Protein-export membrane protein SecG |
| *secY* | U | COG0201 | TIGR00967 | Protein translocase subunit SecY |
| *serS* | J | COG0172 | TIGR00414 | Serine-tRNA ligase |
| *smpB* | O | COG0691 | TIGR00086 | SsrA-binding protein |
| *tig* | O | COG0544 | TIGR00115 | Trigger factor |
| *tilS* | J | COG0037 | TIGR02432 | tRNA(Ile)-lysidine synthase |
| *truB* | J | COG0130 | TIGR00431 | tRNA pseudouridine synthase B |
| *tsaD* | J | COG0533 | TIGR03723 | tRNA N6-adenosine threonylcarbamoyltransferase |
| *tsf* | J | COG0264 | TIGR00116 | Elongation factor Ts |
| *uvrB* | L | COG0556 | TIGR00631 | UvrABC system protein B |
| *ybeY* | J | COG0319 | TIGR00043 | Endoribonuclease YbeY |
| *ychF* | J | COG0012 | TIGR00092 | Ribosome-binding ATPase YchF |

COG category distribution of the 92 genes used in the UBCG analysis (see list above).
