# Supplementary material for: Defining the Species Micromonospora saelicesensis and Micromonospora noduli Under the Framework of Genomics
Source: Front Microbiol. 2018 Jun 25;9:1360. doi: 10.3389/fmicb.2018.01360 (PMC6026663; doi:10.3389/fmicb.2018.01360)
Supplement: Table S5 — Differential phenotypic characteristics between M. saelicesensis and M. noduli as reported by Carro and colleagues (Carro et al., 2016). +, Positive; –, Negative; w, Weak. [file Table_5.DOCX]

**Table S5**. Differential phenotypic characteristics between *M. saelicesensis* and *M. noduli* as reported by Carro and colleagues (Carro et al. 2016)

+, Positive; -, Negative; w, Weak

|  | ***M. saelicesensis* (Group I)** | | | | | | ***intra-species variability M. saelicesensis*** | ***M. noduli* (Group II)** | | | | | | **intra-species variability *M. noduli*** |
| --- | --- | --- | --- | --- | --- | --- | --- | --- | --- | --- | --- | --- | --- | --- |
|  | **Lupac 09^T^** | **Lupac 06** | **GAR05** | **GAR06** | **PSN01** | **PSN13** |  | **GUI43^T^** | **MED15** | **ONO23** | **ONO86** | **Lupac 07** | **LAH08** |  |
| APIZYM |  |  |  |  |  |  |  |  |  |  |  |  |  |  |
| Alkaline phosphatase | + | + | + | + | + | + | 0% | + | w | + | - | + | w | 16.67% |
| Lipase (C 14) | - | w | - | + | - | - | 33.33% | - | - | - | - | - | - | 0% |
| Acid phosphatase | + | + | + | + | + | + | 0% | + | + | + | + | + | + | 0% |
| Naphthol-AS-BI-Phosphohydrolase | + | + | + | + | + | + | 0% | + | + | + | + | + | + | 0% |
| α-galactosidase | + | + | + | + | + | + | 0% | + | + | + | + | + | + | 0% |
| α-glucosidase | + | + | + | + | + | + | 0% | + | + | + | + | + | + | 0% |
| β-glucosidase | + | + | + | + | + | + | 0% | + | + | + | + | + | + | 0% |
| α-mannosidase | - | - | - | - | - | - | 0% | - | - | - | - | - | - | 0% |
| α-fucosidase | - | - | - | - | - | - | 0% | - | - | - | - | - | - | 0% |
| Carbon sources |  |  |  |  |  |  |  |  |  |  |  |  |  |  |
| Alanine | + | + | + | + | + | + | 0% | + | + | + | + | + | + | 0% |
| Arginine | + | + | + | + | + | + | 0% | - | + | + | + | + | + | 16.67% |
| Gluconate | + | + | + | + | + | + | 0% | + | + | + | + | + | + | 0% |
| Histidine | + | + | + | + | + | + | 0% | - | + | + | + | + | + | 16.67% |
| Lysine | - | - | - | - | - | - | 0% | - | - | - | - | - | - | 0% |
| Melezitose | + | + | + | + | + | + | 0% | - | + | + | + | + | + | 16.67% |
| Proline | - | - | - | - | - | - | 0% | - | - | - | - | - | - | 0% |
| Rhamnose | - | - | - | - | - | + | 16.67% | + | + | + | + | + | + | 0% |
| Salicin | + | + | + | + | + | + | 0% | w | + | + | + | + | w | 0% |
| Serine | - | - | - | - | - | - | 0% | - | - | w | w | - | - | 33.33% |
| Sorbose | - | - | - | - | - | - | 0% | - | - | - | - | - | - | 0% |
| Starch | + | + | + | + | + | + | 0% | + | + | + | + | + | + | 0% |
| Trehalose | + | + | + | + | + | + | 0% | + | + | + | + | + | + | 0% |
| Valine | - | - | - | - | - | - | 0% | - | - | - | - | - | - | 0% |
| Xylose | + | + | + | + | + | + | 0% | + | + | + | + | + | + | 0% |
| Maximum NaCl tolerance | 1 | 3 | 3 | 3 | 3 | 3 | 16.67% | 3 | 3 | 3 | 3 | 3 | 3 | 0% |
| pH tolerance | 6-9 | 6.5-9 | 7-9 | 6-9 | 6-9 | 6-9 | 33.33% | 6-9 | 7-9 | 6-9 | 6-9 | 6-9 | 6.5-9 | 33.33% |
| Degradation of |  |  |  |  |  |  |  |  |  |  |  |  |  |  |
| Starch | + | + | + | + | + | + | 0% | + | + | + | + | + | + | 0% |
| Tween 20 | - | - | - | - | - | + | 16.67% | - | - | - | - | - | - | 0% |
| Tween 80 | - | - | - | - | - | + | 16.67% | - | - | - | - | - | - | 0% |
| Tyrosine | - | - | + | - | - | - | 16.67% | + | + | - | + | - | + | 33.33% |
| Urea | - | - | - | - | - | - | 0% | - | - | - | - | - | - | 0% |
